# The potential therapeutic value of the natural plant compounds matrine and oxymatrine in cardiovascular diseases

**DOI:** 10.3389/fcvm.2024.1417672

**Published:** 2024-07-08

**Authors:** Shanjiang Chen, Shu Wu, Bin Lin

**Affiliations:** Department of Cardiovascular Medicine, Wenzhou Central Hospital, Wenzhou, China

**Keywords:** matrine, oxymatrine, cardiovascular disease, pharmacological mechanism, pharmacokinetics

## Abstract

Matrine (MT) and Oxymatrine (OMT) are two natural alkaloids derived from plants. These bioactive compounds are notable for their diverse pharmacological effects and have been extensively studied and recognized in the treatment of cardiovascular diseases in recent years. The cardioprotective effects of MT and OMT involve multiple aspects, primarily including antioxidative stress, anti-inflammatory actions, anti-atherosclerosis, restoration of vascular function, and inhibition of cardiac remodeling and failure. Clinical pharmacology research has identified numerous novel molecular mechanisms of OMT and MT, such as JAK/STAT, Nrf2/HO-1, PI3 K/AKT, TGF-β1/Smad, and Notch pathways, providing new evidence supporting their promising therapeutic potential against cardiovascular diseases. Thus, this review aims to investigate the potential applications of MT and OMT in treating cardiovascular diseases, encompassing their mechanisms, efficacy, and safety, confirming their promise as lead compounds in anti-cardiovascular disease drug development.

## Graphical Abstract

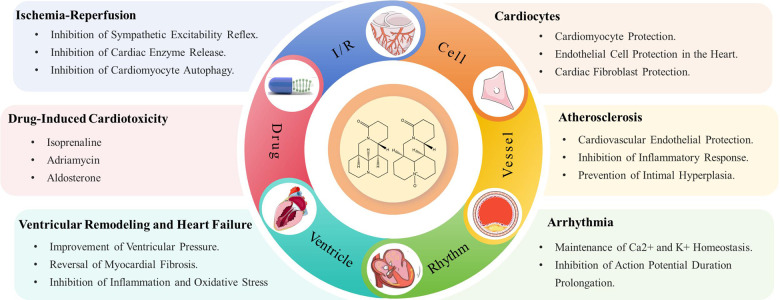

## 1 Introduction

Cardiovascular diseases (CVDs) encompass a range of conditions affecting the heart and vessels (arteries, veins, and capillaries), often manifesting clinically as atherosclerosis, hypertension, heart failure, cardiomyopathy, arrhythmias, and myocardial infarction. With ongoing socioeconomic growth and population ageing, risk factors such as smoking, unhealthy diets, hypertension, dyslipidemia, diabetes, chronic kidney disease, metabolic syndrome, and high stress levels are poised to elevate the risk of CVDs (1, 2). CVDs are among the leading causes of mortality worldwide, with ischemic heart disease being the primary reason for cardiovascular-related deaths in both men and women (3).

To date, research into the mechanisms of cardiac diseases has been ongoing. The mechanisms are complex and interrelated, with different types of heart conditions involving varied pathological processes. Here are some of the primary heart diseases and their associated mechanisms. Atherosclerosis is a fundamental cause of many cardiac conditions. When ox-LDL enters the arteries, its oxidation triggers an inflammatory response, attracting immune cells like macrophages to accumulate and form plaques, causing narrowing or blockage of vessels and limiting blood flow to the myocardium (4, 5). Plaque rupture under strain can initiate the coagulation process, leading to thrombosis, which may rapidly obstruct the supplying arteries, causing myocardial ischemia and, in severe cases, myocardial infarction (6). Reperfusion following ischemia induces oxidative stress and inflammatory responses in cardiac cells, resulting in myocardial cell apoptosis (7). Myocardial ischemia, hypertension, and excessive death of cardiac cells alter the heart's composition and morphology, inducing ventricular remodeling, which may ultimately lead to heart failure (8). These mechanisms may manifest differently across various types of cardiovascular diseases and may interact, contributing to the development and progression of these conditions. Despite numerous therapeutic strategies for cardiovascular diseases, mortality rates remain high. Western medicines, such as statins, anticoagulants, beta-blockers, nitrates, and antithrombotic drugs, are the mainstay treatments for these patients (9). Despite substantial evidence of their efficacy in treating cardiovascular diseases, the potential for serious adverse effects limits the use of these drugs (10, 11). Consequently, there is an urgent need to develop alternative and complementary therapies for better management of cardiovascular diseases.

MT and OMT are natural alkaloids primarily extracted from the Fabaceae family, such as *Sophora flavescens* Ait., *S. tonkinensis* Gagnep, *S. alopecuroides* L., and *S. viciifolia* Hance (12). These medicinal plants have a long history of use in China and many East Asian countries. MT and OMT, due to their clinical efficacy and safety, have been developed into various formulations for clinical use in Asian countries (13, 14). Pharmacological research has identified MT and OMT as some of the most promising natural compounds for treating cardiovascular diseases. To date, there is no comprehensive review of MT and OMT's cardioprotective effects in PubMed or other relevant databases. Therefore, this article provides a timely and in-depth review of the therapeutic potential and molecular targets of MT and OMT in the treatment of cardiovascular diseases, aiming to further advance their development and application.

## 2 The basic characteristics of MT and OMT

**Figure 1:**
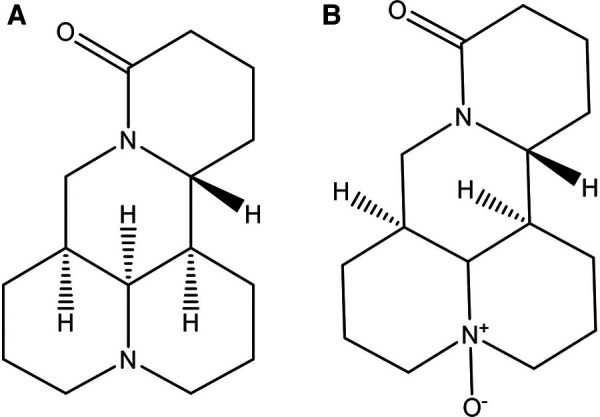
Chemical structures of matrine (**A**) and oxymatrine (**B**).

MT (Figure 1A) is a quinolizidine alkaloid, characterized by two piperidine rings fused by sharing a nitrogen atom. There are two N atoms in the structure, one is tertiary amine N and the other is amide N. The molecular formula of the substance is C_15_H_24_N_2_O, and the molar mass is 248.37. MT pure product is a white powder with a unique bitter taste of alkaloids. The boiling point of the substance is 396.7°C, the melting point is 77°C, it can be dissolved in water, methanol, ethanol, benzene, chloroform and other polar solvents, and is slightly soluble in petroleum ether (15). This compound can be extracted from the roots, fruits, and other parts of its source plants through methods such as extraction and reflux. Notably, it cannot coexist with other alkaline substances; mixing them may trigger chemical reactions, leading to reduced or lost medicinal activity, or even the formation of unstable compounds or hazardous substances (16) (http://www.baidu.com, last accessed on 03/26/2024).

OMT (Figure 1B) is also a quinolizidine alkaloid, with the molecular formula C15H24N2O2 and a molar mass of 264.36. It has a boiling point of 497.19°C and a melting point of 197°C (17). Structurally, OMT is the N-oxide of MT, featuring semi-polar coordination bonds. Consequently, OMT has increased polarity, is more soluble in water and dissolves in chloroform, but is less soluble in solutions like ether and petroleum ether (18). Pure OMT appears brown or deep yellow, with its color deepening upon further oxidation. The extraction method of OMT is the same as that of MT. According to the different polarity, the extract can be extracted by ether, and MT is dissolved in the ether solution to achieve the separation of the two (http://www.baidu.com, last accessed on 03/26/2024).

## 3 Pharmacokinetics of MT and OMT

Pharmacokinetics is pivotal in drug development by elucidating a drug's absorption, distribution, metabolism, and excretion. Clinicians can choose the appropriate formulation, administration route, and dosing frequency based on pharmacokinetic outcomes to ensure maximal therapeutic efficacy. Furthermore, pharmacokinetics can assess metabolic pathways and byproducts, unveiling the drug's mechanism of action. Therefore, to enhance the development potential of MT and OMT as medicinal formulations, it is essential to further explore their absorption and clearance characteristics both *in vivo* and *in vitro*. Current studies suggested that the bioavailability of MT and OMT was relatively low, primarily due to their poor solubility and stability in the gastrointestinal tract, leading to low oral absorption rates. Moreover, they may undergo significant first-pass metabolism in the liver (i.e., extensive metabolism before reaching systemic circulation), further reducing their bioavailability (19, 20).

When MT soft capsules (100, 200, 400 mg) were orally administered in humans, the Tmax (time to reach maximum plasma concentration) was approximately 1.5 h, and the T1/2β (elimination half-life) was around 8 h, with neither Tmax nor t1/2β showing significant variation with dose changes (21). During intravenous infusion (6 mg/ml), the Tmax of MT was approximately 5.6 h, and the T1/2β was 9.5 h (22). When administered orally to SD rats, the absolute bioavailability of MT was approximately 17.1%, with tissue distribution in descending order of liver, spleen, lung, brain, and heart. The concentration in the liver was about 5 times that in plasma. However, MT metabolism did not occur through the liver's most common metabolic pathways, CYP and UGT (23). MT was absorbed through intestinal cells by passive diffusion, showing significant regional differences within the gut. Intestinal permeability was highest in the ileum (Pw = 6.18), followed by the colon (Pw = 2.07), duodenum (Pw = 0.61), and jejunum (Pw = 0.52). MT exhibited linear pharmacokinetics in humans (21, 24). A study indicated that MT inhibited HOCT1-mediated transport by 88% but did not affect hOCT3-mediated transport. This suggested potential drug interactions during hepatic and renal uptake mediated by HOCT1, as well as intestinal absorption through hOCT3 (25). Drug co-administration could alter MT's pharmacokinetics. ACAPHA, an anticancer drug composed of various herbs including *Sophora flavescens* Ait., showed markedly different pharmacokinetic properties compared to the same dose of pure MT. Initially, MT concentrations in ACAPHA were lower than in pure MT; however, 2 h post-administration, MT levels from ACAPHA surpassed those of pure MT in the kidneys, and after 4 h, in plasma and other tissues. The matrix effect and solubility differences within ACAPHA modified MT absorption (26).

Zhang et al. (27) observed that the Tmax of oral OMT (300, 400, 600 mg) in humans was approximately 1.2 h, with a T1/2β around 2.3 h. Additionally, during intravenous infusion (6 mg/ml), OMT's Tmax was 0.5 h, with a T1/2β of 2.2 h. Furthermore, the pharmacokinetic parameters of capsule and dispersible tablet formulations were similar, indicating that the dispersible tablet did not enhance the diffusion rate of OMT (28). After intravenous administration, the concentration-time distribution of OMT followed a two-compartment pharmacokinetic model. Furthermore, both OMT and MT exhibited low plasma protein binding rates (OMT: 4.80%–8.95%, MT: 5.10%–10.55%), ensuring their efficient permeability (22). P-glycoprotein (P-gp) was located on the epithelial cell membrane and was involved in the transport of many drugs. However, numerous absorption experiments based on Caco-2 cells indicated that P-gp may not play a significant role in the transport of OMT. The apparent permeability coefficient (Papp) of OMT in the absorptive direction was 6.39 × 10^−6^ cm/s, which was lower than its Papp value of 11.28 × 10^−6^ cm/s in the secretory direction, with a permeation rate constant of approximately 0.8. Rat intestinal perfusion experiments reveal that the absorption of OMT and MT did not exhibit concentration saturation, and inhibitors of P-glycoprotein, endocytosis, and metabolism did not affect their absorption (29, 30). These findings suggested that P-gp did not participate in the transport of OMT and MT, indicating that their absorption primarily involved passive diffusion mechanisms. The distribution of OMT in tissues followed the order of kidney/lung>muscle>intestine/stomach>heart/spleen>uterus>adipose/testes>brain/liver, which differs from that of MT (31). When OMT was used as a topical formulation, the method of local external application to the skin may not be suitable. Studies showed that during topical application, OMT and MT account for only 1% in the dermis, indicating that the low enzyme content in dermal cells hindered the decomposition of OMT. Therefore, although the skin barrier system impeded the penetration of OMT, the lack of enzymes in dermal cells may be a key factor limiting the efficacy of OMT (32). The primary metabolic pathways for OMT and MT involved renal metabolism, with approximately 50% of the parent drugs excreted in the urine (27). The main metabolites include oxysophocarpine, sophocarpine, 9,10-dihydro-sophocarpine, 3,4,7,8-dihydro-sophocarpine, and 14-hydroxy-matrine (33).

## 4 Application of MT and OMT in cardiovascular diseases

MT and OMT exhibited strong anti-cardiovascular disease effects in both *in vivo* and *in vitro* studies (Tables 1, 2). MT and OMT's cardioprotective effects involved multiple mechanisms. Firstly, they exerted antioxidant actions to reduce oxidative stress, lowering the risk of cardiovascular diseases. Secondly, they had significant anti-inflammatory properties, inhibiting the production of inflammation-related cytokines and mitigating the inflammatory response in cardiovascular conditions. Additionally, they offered protection to patients with cardiovascular diseases by improving vascular function, reducing blood pressure, and inhibiting cardiac remodeling.

**Table 1:** Therapeutic effects of MT in Various cardiac disease models.

Diseases	Model	Dosage	Mode of administration/treatment time	Pharmacological mechanisms	*In vivo*/*vitro*	References
I/R	Left Anterior Descending Coronary Artery Ligation and Reperfusion in Rats	50, 100 mg/kg	Intraperitoneal injection/7 days	MT reduced myocardial infarction size by activating the JAK2/STAT3 pathway and upregulating HSP70 expression.	*In vivo*	Guo et al. (34)
I/R	H/R-induced I/R cardiomyocytes	200 μmol/L	Incubation culture	MT inhibited apoptosis by activating the Sirt3/AMPK pathway.	*In vitro*	Lu et al. (35)
I/R	H/R-induced I/R cardiac microvascular endothelial cells	0.5, 1, 1.5 mg/ml	Incubation culture/2 h	MT suppressed the expression of apoptotic proteins via the JAK2/STAT3 signaling pathway.	*In vitro*	Zhao et al. (36)
Arrhythmia	Ventricular papillary muscle of Wistar rats	10 μmol/L	Incubation culture	MT significantly counteracted homocysteine-induced reductions in both + dt/dt and -dt/dt as well as the inhibitory effects on Ca^2+^, preventing the occurrence of negative inotropy.	*In vitro*	Cai et al. (37)
Arrhythmia	Ouabain-induced arrhythmia in guinea pigs	5, 15, 40 mg/kg	–	MT shortened APD by inhibiting L-type Ca^2+^ currents and reducing Ca^2+^ overload.	*In vivo*	Zhou et al. (38)
Arrhythmia	Rat left anterior descending coronary artery ligation.	30 mg/kg/d	Gastric irrigation/90 days	MT shortened APD and reduced mortality in rats post-myocardial infarction by restoring the expression of Kv4.2/Ito and Kir2.1/IK1 in ventricular myocytes.	*In vivo*	Li et al. (39)
Arrhythmia	Electrical pacing-induced atrial fibrillation in mice.	15, 30, 45 mg/kg/d	Intravenous injection/15 days	MT dose-dependently reduced IKM3 density and upregulated ICa-L density.	*In vivo*	Zhou et al. (40)
Arrhythmia	High-frequency left atrial pacing method for creating a chronic atrial fibrillation rabbit model.	5, 10, 20, 40 mg/kg	Intravenous injection	MT exhibited anti-atrial fibrillation properties by prolonging atrial muscle fAPD and accelerating conduction velocity.	*In vivo*	Tu et al. (41)
Myocardial injury	DOX-induced H9C2 cardiomyocyte injury model.	50, 150, 200 mg/L	Incubation culture/24 h	MT mitigated cardiomyocyte apoptosis by enhancing mitochondrial Na ^+ ^-K ^+ ^-ATPase and Ca2^+^-ATPase activities, thereby improving mitochondrial membrane potential.	*In vitro*	Xu et al. (42)
Myocardial injury	DOX-induced cardiotoxicity in mice and injury in H9C2 cells.	200 mg/kg/d	Gastric irrigation/28 days	MT alleviated oxidative stress and cardiomyocyte apoptosis in cardiotoxicity by maintaining the AMPKα/UCP2 pathway.	*In vivo*/*vitro*	Hu et al. (43)
Myocardial injury	Sepsis-induced myocardial injury in mice.	25, 50 mg/kg/d	Intraperitoneal injection/3 days	MT inhibited cardiomyocyte ferroptosis by activating the PI3 K/AKT pathway.	*In vivo*	Xiao et al. (44)
Myocardial injury	Cecal ligation and puncture-induced septicemia model in mice; lipopolysaccharide-induced injury in H9C2 cells.	200 mg/kg/d; 200 μmol/L	Gastric irrigation/10 days; Incubation culture/24 h	MT enhanced cardiomyocyte viability and attenuated inflammatory responses via the PTENP1/miR-106b-5p axis.	*In vivo*/*vitro*	Liu et al. (45)
Myocardial injury	Rat injection of IOS induces acute myocardial injury.	50, 100, 200 mg/kg/d	Oral administration/10 days	MT restored rat endocardial necrosis and left ventricular dysfunction by inhibiting the expression of target DDAH2 and ADMA through the activation of the Akt/eNOS signaling pathway.	*In vivo*	Li et al. (46)
Myocardial injury	Rat injection of IOS induces acute myocardial injury.	50, 100, 200 mg/kg/d	Oral administration/10 days	MT improved left ventricular dp/dtmax and dp/dtmin, and reduced cardiomyocyte death via its antioxidant action.	*In vivo*	Li et al. (47)
Myocardial injury	High glucose-induced H9C2 cardiomyocyte injury.	1.5 mmol/L	Incubation culture/24 h	MT reduced the expression of inflammatory cytokines IL-1β, IL-6, and TNF-α and enhanced the expression of antioxidant proteins GSH-Px, CAT, and SOD.	*In vitro*	Hu et al. (48)
Diabetic cardiomyopathy	Intraperitoneal injection of AGEs in rats.	50, 100, 200 mg/kg/d	Intraperitoneal injection/20 days	MT mitigates AGE-induced cardiac dysfunction by regulating RyR2-mediated calcium overload.	*In vitro*	Wang et al. (49)
Diabetic cardiomyopathy	Streptozotocin-induced diabetic rat model.	200 mg/kg/d	Oral administration/10 days	MT inhibits cardiomyocyte apoptosis by blocking ROS/TLR-4 signaling through the suppression of ROS production.	*In vivo*	Liu et al. (50)
Diabetic cardiomyopathy	Streptozotocin-induced diabetic rat model.	5 mg/kg/d	Oral administration/70 days	MT exerted anti-inflammatory and antioxidant effects by inhibiting the activation of the TGF-β-induced PERK signaling pathway.	*In vivo*	Hou et al. (51)
Diabetic cardiomyopathy	Rat ventricular myocytes induced by 25 mmol/L high-glucose DMEM	5 mol/L	Incubation culture	MT promoted mitochondrial fusion by activating Mfn2, ameliorating oxidative stress-induced death in cardiomyocytes.	*In vitro*	Xiao et al. (52)
Diabetic cardiomyopathy	Primary peritoneal macrophages.	0.5, 1.0, 1.5, 2.0, 2.5, 3.0 mmol/L	Incubation culture/48 h	MT inhibited oxidative stress by enhancing GPX1 expression and further suppressed M1 cell polarization by inhibiting the activation of the TLR4/STAT1 pathway.	*In vitro*	Cui et al. (53)
Thrombus	Fibrinogen-induced platelet aggregation.	0.25, 0.5, 1.0 mg/ml	Incubation culture/1 h	MT prevented platelet aggregation by inhibiting reactive oxygen species production and promoting VASP phosphorylation.	*In vitro*	Zhang et al. (54)
Cardiac fibrosis	Ang II-induced hyperplastic growth of cardiac fibroblasts	2.0–4.0 mmol/L	Incubation culture/24 h	MT was associated with inhibiting fibroblast proliferation, promoting apoptosis, and suppressing mitosis.	*In vitro*	Li et al. (55)
Cardiac fibrosis	Streptozotocin-induced diabetes in SD rats; fibroblasts cultured in high glucose.	200 mg/kg/d; 0.25, 0.5, 1.0, 1.5, 2.0, 2.5 mmol/L	Oral administration/10 days; Incubation culture/48 h	MT inhibited ECM synthesis by blocking ATF6 signaling in fibroblasts.	*In vivo*/*vitro*	Liu et al. (56)
Cardiac fibrosis	Streptozotocin-induced diabetic rat model.	300 mg/weight	Oral administration/10 days	MT inhibited collagen deposition in cardiomyocytes of diabetic rats by blocking TGF-β1/Smad signaling, preventing fibrosis formation.	*In vivo*	Lu et al. (57)
Cardiac fibrosis	Mice developed pathological cardiac fibrosis through aortic banding surgery or continued isoproterenol injections.	200 mg/kg/d	Oral administration/28 days	MT's attenuation of fibrosis in mice may be associated with the activation of the RPS5/p38 signaling pathway.	*In vivo*	Zhang et al. (58)
Atherosclerosis	AGE-induced HCSMCS.	0.25, 0.5, 1.0 mmol/L	Incubation culture/48 h	MT inhibited the contractile-synthetic phenotype transformation of HCSMCs by suppressing the Dll4-Notch signaling pathway.	*In vitro*	Liu et al. (59)
Atherosclerosis	AGE-induced HCSMCS.	0.25, 0.5, 0.75, 1.0 mmol/L	Incubation culture/48 h	MT suppressed the expression of collagen synthesis-related proteins and collagen deposition by promoting Poldip2/mTOR signaling pathway expression.	*In vitro*	Ma et al. (60)
Atherosclerosis	AGE-induced HCSMCS.	0.5, 1.0 mmol/L	Incubation culture/48 h	MT mitigated the phenotypic transformation of HCCMCs by diminishing the activation of the PERK signal-dependent Dll4-Notch pathway in ERS, reducing the expression levels of GRP78, NICD1, and HES1, and the phosphorylation level of PERK in HCSMCs.	*In vitro*	Zhao et al. (61)
Atherosclerosis	VSMC	5, 10, 15, 20 mg/L	Incubation culture/72 h	MT induced cell cycle arrest at the G1 phase by upregulating the p53/p21 signaling pathway, which inhibited the expression of cyclin D1/cdk4 and cyclin E/cdk2.	*In vitro*	Zhu et al. (62)
Atherosclerosis	Dysregulated blood flow induced VSMC migration.	10, 20, 30, 40 mg/L	Incubation culture/0.5 h	MT inhibited VSMC migration under disturbed flow, in part, by downregulation of the ERK1/2-MLCK signaling pathway.	*In vitro*	Zhu et al. (63)
Atherosclerosis	TNF-α-induced VSMC	10, 50, 100 µg/ml	Incubation culture/72 h	MT inhibited the expression of VCAM-1 and ICAM-1 in TNF-α-stimulated VSMC by suppressing ROS production and the activation of NF-κB and MAPK pathways.	*In vitro*	Liu et al. (64)
Atherosclerosis	AGEs induced apoptosis via oxidative stress in aortic endothelial cells.	0.25, 0.5, 1.0, 2.0, 2.5 mmol/L	Incubation culture	MT inhibited the production of reactive oxygen species by stimulating the activation of the MAPK pathway.	*In vitro*	Liu et al. (65)
Atherosclerosis	Mice fed a high-fat diet; ox-LDL-induced HUVECs.	5, 20, 80 mg/g/d; 5, 20, 80 μmol/L	Oral administration/28 d; Incubation culture/12 h	MT enhanced eNOS activity and the expression of the PI3 K/AKT pathway, alleviating abnormal lipid metabolism and inflammatory responses.	*In vivo*/*vitro*	Zhang et al. (66)
Ventricular remodeling	ISO-induced left ventricular hypertrophy model in rats.	50, 100, 200 mg/kg/d	Gastric irrigation/7 days	MT reduced the expression of growth factors IGF-1 and TGF-β1, improving the fibrous structure of rat myocardial tissue.	*In vivo*	Wei et al. (67)
Heart failure	Coronary artery ligation surgery induced heart failure in rats.	3, 10, 30 mg/kg/d	Oral administration/28 days	MT reduced the upregulation of AR and endothelial nitric oxide synthase proteins following heart failure.	*In vivo*	(u et al. (68)
Heart failure	ISO-induced chronic heart failure in rats.	25, 50, 100 mg/kg/d	Gastric irrigation/7 days	MT ameliorated ISO-induced chronic heart failure by upregulating DDAH2 expression and suppressing cTnI, BNP, and ADMA expression.	*In vivo*	Zhang et al. (69)
Heart failure	ISO-induced heart failure in rats.	10, 2, 40 mg/ml/d	Gastric irrigation/15 days	MT improved cardiac function and myocardial fibrosis in rats by inhibiting the expression of the RhoA/ROCK1 signaling pathway.	*In vivo*	Sun et al. (70)

**Table 2:** Therapeutic effects of OMT in Various cardiac disease models.

Diseases	Model	Dosage	Mode of administration/treatment time	Pharmacological mechanisms	*In vivo*/*vitro*	References
Diabetic cardiomyopathy	Streptozotocin injection and high-fat diet-induced type II diabetes in rats	60, 120 mg/kg/d	Gastric irrigation/28 days	OMT exerted anticancer and anti-apoptotic effects by activating the Nrf2/HO-1 signaling pathway and inhibiting the JAK/STAT signaling pathway.	*In vivo*	Huang et al. (71)
Cardiac fibrosis	Ligation of the left anterior descending coronary artery induces myocardial fibrosis in rats.	25, 50 mg/kg/d	Gastric irrigation/28 days	OMT reduced the left ventricular mass/body weight ratio by inhibiting the TGF-β1/Smads signaling pathway.	*In vivo*	Shen et al. (72)
Cardiac fibrosis	Aldosterone stimulated the proliferation of cardiac fibroblasts.	0.757, 0.378 mmol/L	Incubation culture/2 h	OMT reduced the phosphorylation of Smad-2, Smad-3, and Smad-4.	*In vitro*	Fu et al. (73)
Cardiac fibrosis	TGF-β1 induced proliferation and aberrant differentiation of cardiac fibroblasts	0.189, 0.370, 0.756 mmol/L	Incubation culture/1 h	OMT inhibited TGF-β1-induced CFB proliferation, α-SMA elevation, and type I and III collagen deposition via ERK1/2/MAPK pathway suppression.	*In vitro*	Xu et al. (74)
Myocardial injury	Ligation of the left anterior descending coronary artery to establish a rat model of acute myocardial infarction.	25 mg/kg/d	Gastric irrigation/14 days	OMT reduced levels of lactate dehydrogenase, troponin, and pro-brain natriuretic peptide, diminishing myocardial infarction size.	*In vivo*	Ma et al. (75)
Myocardial injury	Aldosterone-induced cardiomyocyte damage.	0.180, 0.378 mmol/L	Incubation culture/2 h	OMT inhibited the expression of apoptotic proteins by suppressing the phosphorylation of JNK protein.	*In vitro*	Yang et al. (76)
Myocardial injury	High glucose-induced oxidative stress damage in H9C2 cells.	50, 100 mg/L	Incubation culture/1 h	OMT significantly reduced lactate dehydrogenase leakage, decreases ROS and MDA levels, and increased SOD activity, mitochondrial membrane potential, and the expression of Bcl-2 protein.	*In vitro*	Yang et al. (77)
Myocardial injury	Intraperitoneal injection of lipopolysaccharide was used to establish a mouse model of myocardial injury.	25, 50 mg/kg	Gastric irrigation/8 days	OMT reduced cardiac tissue inflammatory cell infiltration, serum inflammatory cytokine levels, and the expression of key pyroptosis proteins (NLRP3, Cleaved Caspase-1, GSDMD, GSDMD-N, IL-18, IL-1β).	*In vivo*	Gao et al. (78)
Myocardial injury	Sepsis-induced rat myocardial injury.	13, 26, 52 mg/kg/d	–	OMT mitigated rat heart rate and left ventricular end-diastolic pressure, and enhanced mean arterial pressure, left ventricular pressure change rate, and left ventricular end-systolic pressure by inhibiting the TNF-α/MAPK/caspase-3 inflammatory signaling pathway.	*In vivo*	Zhang et al. (79)
Myocardial injury	Aldosterone-induced rat cardiomyocytes	25 μg/ml	Incubation culture/1 h	OMT prevented apoptosis by suppressing the overexpression of calpain and AIF induced by ALD.	*In vitro*	Xiao et al. (80)
Atherosclerosis	ox-LDL-induced HUVEC injury.	2, 4, 8 µmol/L	Incubation culture/1 h	OMT exerted anti-inflammatory and antioxidant effects by activating the SIRT1/Nrf2 signaling pathway, inhibiting the overexpression of inflammatory cytokines IL-1β and IL-18, and enhancing the activities of SOD, CAT, and GSH-Px.	*In vitro*	Lu et al. (81)
Arrhythmia	Ligation of the left anterior descending coronary artery to establish a rat model of acute myocardial infarction resulted in arrhythmias.	100 mg/kg/d	Gastric irrigation/14 days	OMT upregulated the expression and increased the phosphorylation level of Cx43 in the myocardial tissue surrounding the infarct, reducing the incidence of ventricular arrhythmias.	*In vivo*	Qin et al. (82)
Ventricular remodeling	Lycorine induced right ventricular hypertrophy in rats with pulmonary arterial hypertension.	25, 50 mg/kg	Gastric irrigation/28 days	OMT reduced the protein expression of RhoA, ROCK1, and ROCK2 in right ventricular tissue and the ratio of right ventricular mass to left ventricular plus septal mass.	*In vivo*	Li et al. (83)
Ventricular remodeling	Ligation of the left anterior descending artery to establish an acute myocardial infarction model in rabbits.	1.0 ml/100 g	Gastric irrigation/28 days	OMT improved cardiac output, left ventricular remodeling parameters, and cardiac function in rabbits post-acute myocardial infarction.	*In vivo*	Wang et al. (84)
Ventricular remodeling	Coxsackievirus B3 induced ventricular remodeling in heart failure mice.	13, 26, 52 mg/kg/d	Subcutaneous injection/14 days	OMT reverses ventricular remodeling by protecting mitochondria, enhancing myocardial energy metabolism, and reducing cardiomyocyte apoptosis.	*In vivo*	Wang et al. (85)
Heart failure	ISO-induced heart failure in rats.	25, 50, 100 mg/kg/d	Gastric irrigation/7 days	OMT treatment markedly decreased serum brain natriuretic peptide and troponin I level, and lowered B7–2 co-stimulatory molecules and neutrophil chemokines (CINC-2, CINC-3) expression in cardiac tissue.	*In vivo*	Xu et al. (86)
Heart failure	ISO-induced heart failure in rats.	25, 50, 100 mg/kg/d	Gastric irrigation/7 days	OMT ameliorated left ventricular hypertrophy and dysfunction in heart failure rats by modulating the DDAH/ADMA metabolic pathway.	*In vivo*	Zhang et al. (87)
Heart failure	ISO-induced heart failure in rats.	25, 50, 100 mg/kg/d	Oral administration/14 days	OMT significantly reduced the increase of BNP in rat plasma, mitigated cardiac fibrosis, inhibited the overexpression of myocardial COX-1, and upregulated the expression of COX-2 and PGIS.	*In vivo*	Zhang et al. (88)
Heart failure	ISO-induced heart failure in rats.	25, 50, 75 mg/kg/d	Gastric irrigation/28 days	OMT significantly suppressed the levels of inflammatory cytokines TNF-α and IL-6. It reduced the level of TLR4 and decreased the phosphorylation levels of IκB, p65, JNK, and p38.	*In vivo*	Sun et al. (89)

### 4.1 Ischemic injury

Myocardial ischemia, caused by coronary artery narrowing, blockage, and its complications, can lead to severe conditions such as angina, myocardial infarction, and even sudden death. Arteriosclerosis and diabetes are among the factors that can induce myocardial ischemia. It is a leading cause of death and disability worldwide (90). During myocardial ischemia, the excitatory reflex of the sympathetic nervous system further increases myocardial oxygen consumption, exacerbating the adverse effects of myocardial ischemia (91). The primary regulatory neuroarc in nociceptive transmission during myocardial ischemia involved the SCG and DRG, marked by the overexpression of P2X3 receptors in sympathetic neurons (92). OMT reduced the expression of P2X3 receptors, inhibiting the exacerbation of sympathetic excitatory reflexes caused by P2X3 receptor-mediated nociceptive transmission in rat SCG and DRG neurons during myocardial ischemic injury (93, 94).

Ischemia-reperfusion (I/R) was a common intervention for myocardial ischemia, yet it could exacerbate cardiomyocyte death through various mechanisms, including oxidative stress, calcium overload, activation of pro-apoptotic pathways, and inflammatory responses (95, 96). However, clinically, few drugs effectively protected cardiomyocytes from I/R injury. Thus, investigating the potential mechanisms of MT and OMT on I/R could aid in mitigating its damage. During I/R, myocardial membrane integrity was compromised, resulting in the release of cardiac enzymes such as cTnI, CTnT, LDH, and CK-MB. OMT treatment significantly reversed these effects (97). I/R induced autophagy in cardiomyocytes, characterized by increased expression of autophagic markers LC-3II/LC-3I, Beclin-1, and Atg5. OMT reduced these responses and suppressed levels of inflammatory cytokines TNF-α, IL-6, and IL-8, maintaining autophagic homeostasis in cardiomyocytes (98). Another manifestation of I/R was the induction of cellular endoplasmic reticulum stress (ERS). Under ERS conditions, the expression of PERK, IRE1α, and ATF6 significantly increased, contributing to ROS production and apoptosis. Treatment with MT notably reduced the levels of these three markers in the peripheral blood of rats (97).

**Figure 2:**
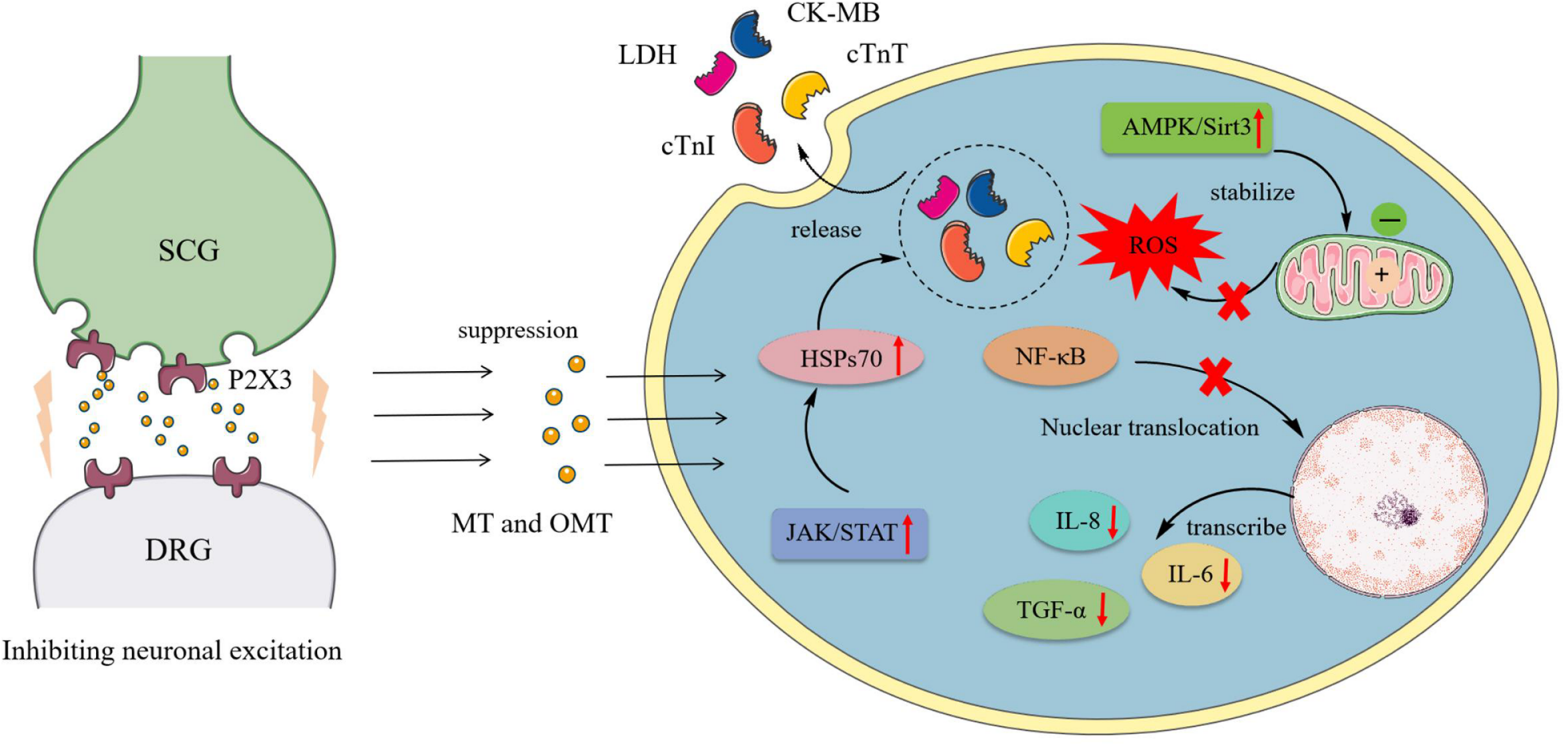
Pharmacological mechanism of MT and OMT against myocardial ischemia. SCG, Superior cervical ganglia; DRG, Dorsal root ganglia; LDH, Lactate dehydrogenase; CK-MB, Creatine kinase-MB; CTnT, Cardiac troponin T; CTnI, Cardiac troponin I; HSPs70, Heat shock proteins 70.

Furthermore, the JAK/STAT pathway was closely associated with I/R, where STAT family proteins were directly linked to apoptotic behavior (99). Downstream factor HSP70, proved to be a cardioprotective agent, could inhibit cardiomyocyte autophagy and prevent apoptosis upon activation (100, 101). MT, acting as a JAK/STAT agonist, simultaneously activated this pathway and enhanced HSP70 expression in I/R cardiomyocytes, reducing lactate dehydrogenase release, creatine kinase activity, and *in vitro* cardiomyocyte apoptosis. These effects are abrogated by the JAK2 inhibitor AG490 and HSP70 siRNA (34). Zhou et al. (102) identified high affinity and stable hydrogen bonding between MT and JAK2 through bioinformatics. Further investigations revealed that MT promotes the phosphorylation of JAK2 and STAT3 and the expression of anti-apoptotic proteins Bcl-xL and Bcl-2 in I/R cells. Additionally, activation of the AMPK/Sirt3 pathway constituted one of MT's mechanisms influencing I/R, where pathway upregulation revitalized cardiomyocytes, stabilized mitochondrial membrane potential, suppressed excessive oxidative reactions, and mitigated I/R-induced cardiac injury (35). Figure 2 shows the pharmacological mechanism of MT and OMT against myocardial ischemia.

### 4.2 Cell damage

**Figure 3:**
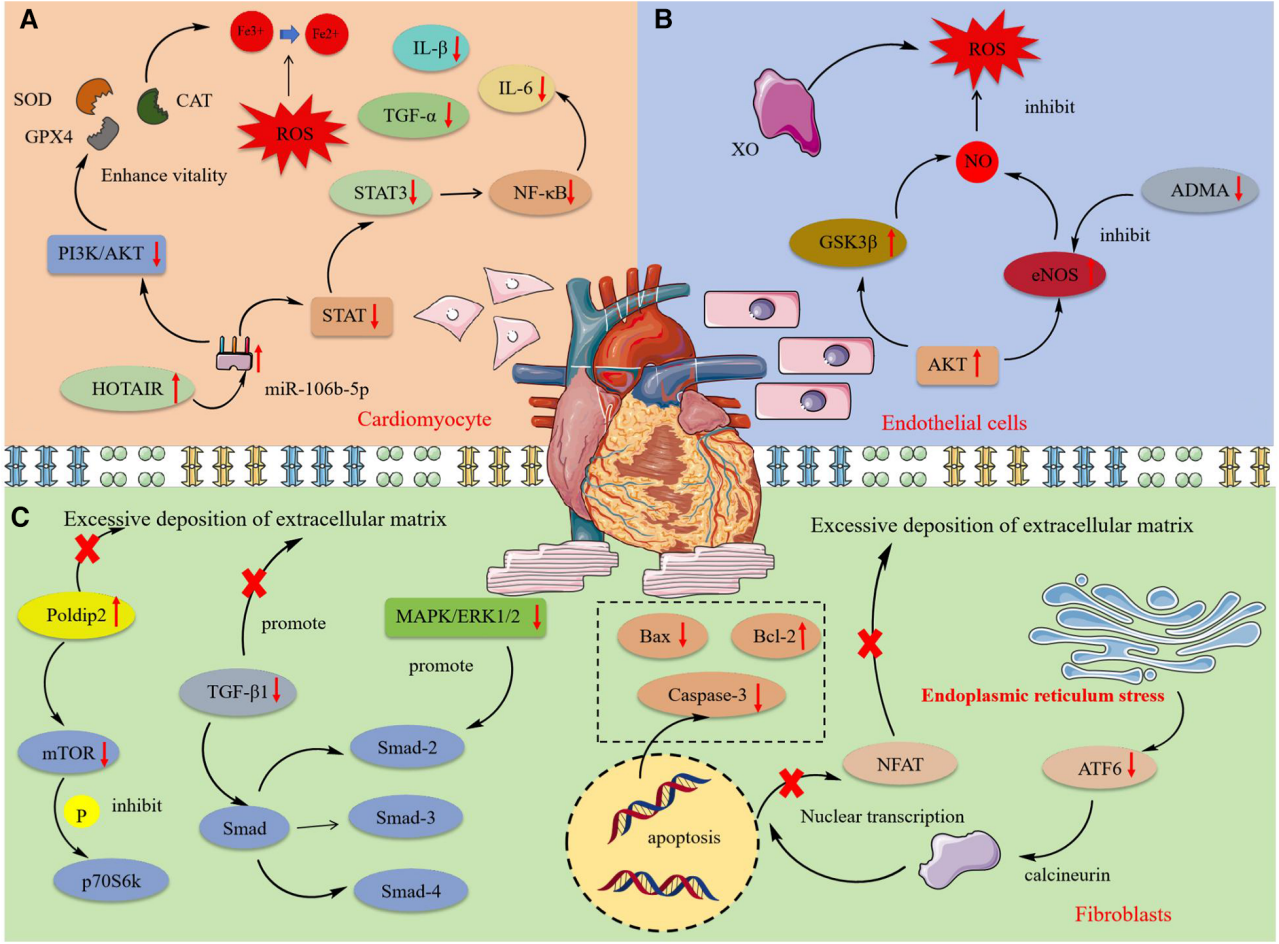
The mechanism of protective effect of MT and OMT on cardiac cells. SOD, Superoxide dismutase; CAT, Catalase; GPX4, Glutathione peroxidase 4; STAT, Signal transducer and activator of transcription; HOTAIR, HOX transcript antisense RNA; XO, Xanthine oxidase; Gsk3β, glycogen synthase kinase 3β; ADMA. Asymmetric dimethylarginine; eNOS, Endothelial nitric oxide synthase; Poldip2, polymerase delta interacting protein 2; p70S6 K, 70 kDa Ribosomal Protein S6 Kinase 2; ATF6, Activating transcription factor 6.

Cardiomyocytes encompass various functional cells such as myocardial cells, endothelial cells, and fibroblasts, whose synergistic actions sustain cardiac function. As non-regenerable terminally differentiated cells, their damage leads to irreversible cardiac dysfunction, thus triggering a range of cardiovascular diseases including angina, arrhythmias, and heart failure (103). Investigating the mechanisms of cardiomyocyte damage and identifying effective therapeutic drugs are crucial for both the theoretical and clinical management of cardiovascular diseases. Figure 3 shows the protective mechanism of MT and OMT on cardiac cells.

#### 4.2.1 Myocardial cell injury

Myocardial cell damage was closely associated with oxidative reactions and inflammation. High glucose levels could lead to myocardial cell death, with oxidative stress and inflammatory response being key processes in its damage (104, 105). MT enhanced GSH-PX and CAT activity in H9C2 cardiomyocytes under high glucose or H2O2 conditions and inhibited the activation of inflammatory factors IL-1β, IL-6, and TNF-α mediated by the NF-κB inflammatory pathway, thereby suppressing oxidative and inflammatory responses in cardiomyocytes (48, 106). Experimental evidence suggested this effect may be related to the intervention in the expression of long non-coding RNA HOTAIR. In H9C2 cells under oxidative conditions, HOTAIR expression was significantly reduced, which was effectively reversed by MT treatment. HOTAIR negatively regulated the synthesis of miR-106b-5p, further inhibiting the activation of the PI3 K/AKT and STAT3 pathways, and thus preventing the production of reactive oxygen species and inflammatory factors (107). Additionally, the production of peroxides and lipid peroxidation led to the accumulation of iron ions, further stimulating the generation of reactive oxygen species and resulting in ferroptosis. This regulatable form of cell death was primarily influenced by GPX4 (108). Interestingly, recent studies increasingly report that ferroptosis played a significant role in the onset and progression of cardiovascular diseases. MT could reverse the peroxidation phenomenon in the process of cardiomyocyte ferroptosis, enhance SOD activity in cells, and promote GPX4 expression. This process coincided with the phosphorylation of p-PI3 K, PI3 K, p-AKT, AKT proteins and a decrease in the Bax/Bcl-2 ratio, confirming the PI3 K/AKT pathway as the upstream pathway through which MT inhibited ferroptosis and apoptosis (44). Additionally, OMT could improve mitochondrial membrane potential, inhibit the expression of pro-apoptotic proteins Caspase-3 and Bax, reduce inflammatory cell infiltration in cardiac tissue, and alleviate cardiomyocyte pyroptosis induced by high glucose and lipopolysaccharides (77, 78).

#### 4.2.2 Cardiac endothelial cells

Cardiac endothelial cell damage was associated with excessive oxidative reactions. The production of reactive oxygen species mediated by the myocardial defense system, depletion of antioxidants, and imbalance of nitric oxide often led to oxidative stress responses in endothelial cells, further inducing mitochondrial dysfunction and damage to functional macromolecules, ultimately resulting in cell death (109). Increased XO activity was a significant factor in endothelial oxidative stress, with overexpression triggering the release of ROS (110). Studies confirmed that MT could improve endothelial dysfunction by regulating the ROS/NO balance. MT treatment restored the expression of Akt and eNOS in damaged ventricular cells induced by isoproterenol (ISO), reactivated glycogen synthase kinase 3b, reduced the levels of the eNOS inhibitor ADMA in serum, and maintained NO homeostasis (46).

#### 4.2.3 Cardiac fibroblasts and fibrosis

Cardiac fibroblasts play a crucial role in maintaining the heart's basic structure and function and regulating cardiac remodeling under pathological conditions. During cardiovascular diseases, cardiac fibroblasts proliferate, are activated, and differentiate into contractile myofibroblasts. This differentiation leads to functional changes, including increased proliferation, the release of signaling molecules, and excessive accumulation of the extracellular matrix (ECM), ultimately resulting in cardiac fibrosis (111). Studies had demonstrated that MT and OMT significantly inhibited the malignant proliferation of cardiac fibroblasts. MT exhibited a dose-dependent inhibition of fibroblast proliferation induced by AngⅡ, with most cells arrested in the G1 phase of the cell cycle, a decrease in the Bcl-2/Bax ratio, and an increase in cleaved-caspase-3 activity, further promoting apoptosis of cardiac fibroblasts (55). TGF-β1 was a potent fibrogenic factor critical for the deposition of the extracellular matrix, particularly in the accumulation of type I and III collagen (112). Evidence indicated that MT and OMT significantly reduced the expression of TGF-β1 mRNA, preventing the entry of its downstream target Smad transcription complex (Smad-2, Smad-3, and Smad-4) into the nucleus to bind with specific target genes. This action inhibited collagen deposition in cardiac tissue, restored left ventricular function and compliance, and markedly improved ventricular remodeling in rats (57, 72, 73). Xu et al. (74) discovered that TGF-β1-induced hyperproliferation of cardiac myofibroblasts was associated with significant increases in phosphorylation of the MAPK/ERK1/2 pathway. Interestingly, this effect could be notably suppressed by both MAPK/ERK1/2 inhibitors and OMT. The MAPK/ERK1/2 pathway may be one of the mechanisms through which MT treats fibrosis. Additionally, experiments confirmed that activation of p38 in fibroblasts was sufficient to drive myofibroblast formation and collagen synthesis (113). MT could inhibit p38 activation by upregulating RPS5 expression, mitigating fibrosis remodeling and cardiac dysfunction induced by AB operation or ISO (58).

Studies had linked ERS to diabetic-induced cardiomyopathy fibrosis (114). Inducing ERS activated ATF6, which in turn activated calcineurin. This activated calcineurin engages in the nuclear transcription of the ECM gene expression inducer NFAT, leading to ECM build-up (115). Western blotting revealed that MT attenuated fibroblast calcium accumulation and NFAT activation by inhibiting the ATF6 signaling pathway, suppressing ECM synthesis, and restoring impaired cardiac compliance and function (56). Human coronary artery smooth muscle cells; (HCSMCs) exposed to advanced glycation end products (AGEs) were prone to phenotypic transformation, leading to increased ECM deposition (116). This mechanism was primarily characterized by reductions in MYH11 and ACTA2. Treatment with MT inhibited Notch signaling activation by suppressing the expression of Notch ligand DLL4, increased MYH11 and ACTA2 in HCSMCs, and reversed the AGEs-induced contractile-synthetic phenotypic transformation in HCSMCs (59). Additionally, Ma et al. (60) demonstrated that MT inhibited ECM accumulation by promoting Poldip2 expression, suppressing downstream mTOR protein expression, preventing p70S6k phosphorylation and nuclear translocation, a mechanism confirmed through Poldip2-specific inhibitory siRNA. Overall, MT and OMT effectively inhibited cardiac fibrosis, importantly involving multiple targets and pathways, highlighting their broad and diverse potential in fibrosis treatment. In summary, MT and OMT represent promising natural lead compounds for treating cardiac fibrosis.

### 4.3 Vascular cell injury and atherosclerosis

**Figure 4:**
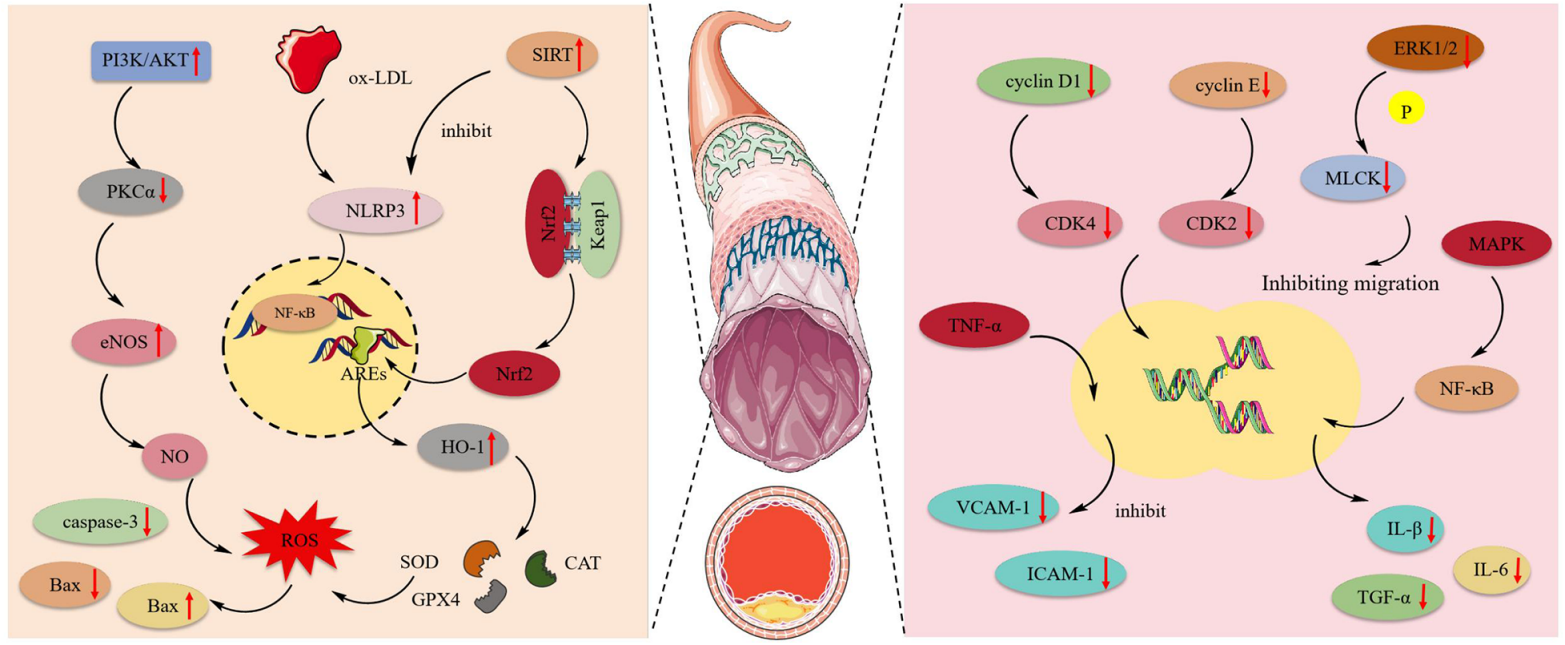
The mechanism of protective effect of MT and OMT on vascular cells. eNOS, Endothelial nitric oxide synthase; NLRP3, NLR family pyrin domain containing 3; ox-LDL, Low-density lipoprotein; HO-1, heme oxygenase-1; Nrf2, Transcription of nuclear factor erythroid 2-like 2; SOD, Superoxide dismutase; CAT, Catalase; GPX4, Glutathione peroxidase 4; MAPK, mitogen-activated protein kinase.

Atherosclerosis is an inflammatory disease and a leading cause of CVDs and stroke. The primary pathology occurs in blood vessels, characterized by the prolonged accumulation and transformation of lipids, inflammatory cells, smooth muscle cells, and necrotic cell debris beneath the endothelial monolayer in the intimal space, ultimately leading to cell damage (117). Figure 4 shows the mechanism of protective effect of MT and OMT on vascular cells.

#### 4.3.1 Vascular endothelium injury

MT had been shown to possess anti-apoptotic effects. By activating the JAK2/STAT3 pathway, it could reduce the expression of caspase-3 and Bax while increasing Bcl-2 expression in cardiac microvascular endothelial cells (CMECs). This promoted cell entry into the S phase and accelerated maturation (36). Human umbilical vein endothelial cells (HUVEC) exposed to ox-LDL were prone to activating NLRP3 inflammasome expression, inducing inflammation and oxidative reactions, leading to focal death. Notably, OMT could activate the SIRT pathway, inhibiting the expression of NLRP3, IL-1β, and IL-18. Additionally, the expression of the downstream target Nrf2 was also enhanced, which could suppress NLRP3-mediated focal death (118). Additionally, this target acts as a critical antioxidant factor, particularly in regulating HO-1 activation. It enhances the activity of SOD, CAT, and GSH-Px in HUVEC while suppressing the production of ROS and MDA (119). This suggests that the SIRT1/Nrf2 signaling pathway is an effective route through which OMT inhibits NLRP3 inflammasome-mediated pyroptosis (81). Furthermore, Zhang et al. (66) observed that ox-LDL disrupts the NO balance in HUVEC, leading to oxidative stress-induced endothelial cell death. MT, by activating the PI3 K/AKT pathway, stimulated the expression of downstream substrate eNOS and inversely inhibits the activation of the eNOS negative regulator PKCα, ultimately increasing NO synthesis. In the aortic endothelial cell apoptosis model induced by AGEs, MT activates the MAPK enzymes MKK3 and MKK6, promoting MAPK phosphorylation. This leads to the nuclear translocation of Nrf2 and its binding to ARE, triggering the transcription of antioxidant enzyme genes. Consequently, it suppresses the production of reactive oxygen species and inhibits endothelial cell apoptosis (65).

#### 4.3.2 Aortic smooth muscle cell injury

Post-aortic injury commonly presented with intimal hyperplasia and luminal stenosis. This occurs due to the pathological stimulation or damage of blood vessels, where Vascular Smooth Muscle Cells (VSMCs) become abnormally activated. The expression of their differentiation marker genes was downregulated, leading to a de-differentiated state with enhanced proliferation and migration capabilities. This resulted in abnormal intimal proliferation, a key process contributing to the development and progression of various cardiovascular diseases such as atherosclerosis and pulmonary arterial hypertension (120, 121). MT could block the cell division cycle, which required the expression and activation of cyclins and CDK complexes. Briefly, the activation of cyclin D1/CDK4 and cyclin E/CDK2 drove cells into the G1 phase and phosphorylated the Rb complex, facilitating entry into the S phase (122). MT inhibited the expression of p53 and p21 in VSMCs, downregulated the levels of cyclin D1/CDK4, cyclin E/CDK2, and the phosphorylated Rb protein, thereby blocking the VSMC G1 phase (62). In addition to its anti-proliferative effects, MT also inhibited the migration of vascular smooth muscle cells under conditions of disturbed blood flow by suppressing the phosphorylation of ERK1/2 and MLCK (123). Atherosclerosis is a chronic inflammatory disease where the overexpression of adhesion molecules in vascular smooth muscle cells can lead to its onset (124). MT inhibited the expression of VCAM-1 and ICAM-1 in human aortic smooth muscle cells (HASMCs) induced by TNF-α. Moreover, post-MT treatment, there was significant suppression of the NF-κB inflammatory pathway and the MAPK pathway, along with a notable decrease in the levels of IL-1β, IL-6, TNF-α, and ROS. This indicated that MT mitigated atherosclerosis by reducing the expression of adhesion molecules in HASMCs through the inhibition of the NF-κB inflammatory pathway, MAPK pathway, and ROS production (64).

### 4.4 Ion balance and arrhythmias

Action potential duration (APD) was a key determinant of arrhythmias, influenced by various ion channel currents and transporters. The transient outward potassium current (Ito) serves as the primary repolarizing current in cardiac cells, and its amplitude reduction could lead to APD prolongation (125). The cardiac IK1 current was a strongly inwardly rectifying K^+^ selective current crucial for shaping the normal action potential, responsible for initial depolarization and final repolarization (126). Prolongation of APD further triggered Ca^2+^ influx and sarcoplasmic reticulum (SR) Ca^2+^ release, leading to cytotoxic Ca^2+^ overload. In response, cardiomyocytes may initiate protective mechanisms by downregulating mRNA and proteins associated with Ca^2+^ channels and accelerating their inactivation to reduce Ca^2+^ influx (127, 128). However, excessive activation of this response led to the clustered downregulation of L-type Ca^2+^ channels (ICa-L) in the atria, paradoxically increasing the risk of ventricular arrhythmias without showing beneficial effects on atrial fibrillation (129). Long-term administration of oral MT in rats with arrhythmias had demonstrated a reduced likelihood of myocardial infarction, restoring the expression of Ito/Kv4.2 and IK1/Kir2.1 proteins in K + channels. Moreover, there was a significant decrease in IKM3 density and an increase in ICa-L density, suggesting that MT ensured normal action potential duration in cardiomyocytes by maintaining the stability of myocardial K^+^ and Ca^2+^ channels, thereby alleviating arrhythmias (39, 40, 130). Ventricular remodeling following myocardial infarction often triggered ventricular arrhythmias, with reduced expression of Cx43 being a primary cause. Studies indicated that post-OMT treatment, the expression and phosphorylation levels of Cx43 in rat myocardial tissue significantly increased, effectively treating myocardial injury and arrhythmias (82).

The balance of intracellular Ca^2+^ concentration was vital for maintaining cardiac viability, affecting not only the rhythm balance as previously discussed but also closely associated with mitochondrial function, oxidative reactions, and apoptosis. Detrimental stimuli induced Ca^2+^ influx, altering mitochondrial membrane potential (MMP) and triggering caspase cascade activation, leading to cell death (131). Overexpression of RyR2 led to the efflux of endoplasmic calcium. MT maintained MMP stability and mitigated mitochondrial damage by enhancing the binding of FKBP12.6 to RyR2, reducing RyR2 activity (49). OMT reduced calpain activity and the content of its target tBID, diminished cytosolic Ca^2+^ concentration, inhibited cytochrome c release from mitochondria to the cytosol and caspase-3 activation, and prevented AIF translocation to the nucleus, thereby decreasing apoptosis (80).

### 4.5 Relieving heart failure

Heart failure represents the end stage of cardiac disease, with nearly all cardiovascular conditions ultimately leading to its onset. Clinically, heart failure is categorized into acute and chronic forms based on the rate of onset (132). Multiple studies have demonstrated the therapeutic potential of MT in animal models of heart failure. In a rat model of chronic heart failure established by ligation of the coronary artery, MT was found to enhance myocardial contractile function by reducing serum levels of AST, CPK, and LDH, and by inhibiting the β3-AR/eNOS pathway (68). In a rat model of heart failure induced by ISO, treatment with OMT and MT effectively improved ventricular dysfunction and myocardial hypertrophy, demonstrating inhibition of ventricular remodeling, oxidative stress, and inflammation biomarkers. Specifically, MT reduced serum levels of BNP (a heart failure biomarker), and facilitated COX-2 and Prostacyclin synthase coupling, thereby converting arachidonic acid into PGI2 (a cardioprotective factor) (88, 133, 134). Sui et al. (70) suggested that the cardioprotective effect of MT against heart failure might be associated with the inhibition of the RhoA/ROCK1 signaling pathway. Inhibition of this pathway significantly improved cardiac function and reduced myocardial fibrosis in rats. OMT attenuated inflammation by suppressing TLR4 levels, inhibiting the nuclear transcription activity of NF-κB, and deactivating the MAPK pathway, thereby reducing serum levels of TNF-α and IL-6 (89). ADMA, an inhibitor of NOS, promoted apoptosis, oxidative stress, and inflammation, and was a significant biomarker of heart failure (135). Post-treatment with OMT (25, 50, and 100 mg/kg for 14 days) in ISO-administered rats enhanced the expression of DDAH2, facilitating the degradation of ADMA, thereby reducing the levels of cardiac biomarkers BNP and cTn-I (69, 87). Additionally, OMT dose-dependently decreased the expression levels of neutrophil chemoattractant factors (CINC-2, CINC-3), thereby reducing inflammation (86).

### 4.6 Inhibiting ventricular remodeling

Ventricular remodeling refers to the pathological and physiological response involving changes in size, shape, wall thickness, and tissue structure of the ventricles, secondary to myocardial injury or increased load. This process is primarily associated with excessive ventricular pressure load, cardiomyocyte hypertrophy, myocardial thickening, fibrosis, and hemodynamics (136). In the aforementioned section, we discussed the therapeutic effects of MT and OMT on myocardial fibrosis, which further prevents the occurrence of ventricular hypertrophy. In animal models, MT and OMT have been shown to reduce the degree of ventricular remodeling. For instance, in acute myocardial infarction rabbit models, MT and OMT (1 mg/100 g/d, for 4 weeks) increased cardiac output, peak rate of left ventricular pressure change (dp/dtmax), and left ventricular end-systolic pressure (LVESP), while decreasing left ventricular end-diastolic pressure (LVEDP), thus stabilizing ventricular pressure (84). OMT (200 mg/kg/d) inhibited the decrease in dP/dtmax and the weight of the heart and left ventricle in rats undergoing cardiac remodeling, protecting the mitochondrial structure. Mechanistic studies revealed that MT suppressed the expression of IGF-1, TGF-β1 proteins, and mRNA, decreased the expression of FFA, LAC, Col I, Col III, GRP78, CHOP, and osteopontin, increased the ATP/AMP ratio and calreticulin expression, thereby reducing myocardial energy metabolism and decreasing cardiomyocyte apoptosis (67, 85). Existing research indicated that Angiotensin II (Ang II), the final active product of the Renin-angiotensin-aldosterone system (RAAS), was involved in cardiac remodeling by regulating cardiac contractility, cellular coupling, and electrical impulse propagation (137). Post-OMT treatment, there was a reduction in ACE expression in the myocardial tissue of hypertensive rats, thus decreasing the conversion of Ang I to Ang II and the deposition of type I and III collagen, alleviating ventricular remodeling (138). Phosphorylation of RhoA GTPase and its downstream ROCKs played a role in processes such as vascular smooth muscle contraction, stress fibre formation, cell migration, and blood pressure regulation (139, 140). OMT exerted an inhibitory effect on the RhoA/ROCK pathway, reducing the formation of adhesion factors and tension fibres, thus alleviating increased cardiac hypertrophy index and right ventricular remodeling in rats with monocrotaline-induced pulmonary arterial hypertension (83, 141).

### 4.7 Diabetic cardiomyopathy

The maintenance of blood glucose homeostasis relies on hepatic gluconeogenesis and glycogenolysis, as well as glucose uptake and utilization by peripheral tissues, facilitated by insulin. Insufficient insulin secretion can lead to hyperglycemia, resulting in diabetes (142). The global mortality rate of diabetes has rapidly increased in recent years, primarily due to its severe cardiovascular complications (114). Recently, natural compounds extracted from herbs have been found to have significant anti-diabetic cardiomyopathy effects, gaining widespread attention for their efficacy, lower side effects, and low cost (143). Recent studies showed that MT and OMT modulated various signaling pathways in diabetic heart disease, making them promising drugs for treating diabetic cardiomyopathy. In *in vivo* models, MT and OMT regulated oxidative stress and inflammation in DCM rats, offering protection. OMT reduces fasting blood glucose, GHb, NEFA, TC, TG, and LDL-c levels in diabetic rats while increasing serum insulin, liver and muscle glycogen, HDL-c, GLP-1, and muscle GLUT-4 content. Histological examination of the pancreas and liver shows that OMT protects islet structure and prevents liver disorganization (144). MT treatment inhibited the expression of two peroxisome proliferator-activated receptors (PPARβ, PPARγ1), and reduced ROS and MDA levels in cardiomyocytes of DCM rats, thus suppressing the activation of the TLR-4/MyD-88 pathway induced by oxidative stress, and decreased the expression of pro-apoptotic factors caspase-8 and caspase-3 (50, 51). MT attenuated oxidative stress by upregulating the expression of Nrf2 and HO-1 (52). Additionally, the peroxidation reaction in DCM rat cells played a critical role in the formation of atherosclerotic plaques by mediating pathological processes in related cell types such as vascular smooth muscle cells, endothelial cells, and macrophages (145). Oxidative stress was a primary trigger for the release of pro-inflammatory cytokines (146). MT inhibited the activation of the TLR4/STAT1 signaling pathway induced by AGEs/RAGE-mediated oxidative stress, promoted the expression of GPX1, and thereby suppressed M1 macrophage polarization (53). Furthermore, MT exerted potential anti-inflammatory effects by downregulating the TGF-β-induced PERK signaling pathway and suppressing levels of inflammatory factors TNF-α and IL-6 (51). Mitochondrial fission observed in DCM rat cardiomyocytes, which accelerated mitochondrial autophagy, was mitigated by MT through activation of Mfn2, facilitating the fusion of damaged mitochondria to preserve function (52). Furthermore, OMT, by activating the Nrf2/HO-1 pathway and inhibiting the JAK/STAT pathway, enhanced the activity of antioxidant enzymes such as glutathione, SOD, and catalase, and modulating the expression of inflammatory factors, thereby alleviating changes in blood glucose levels and left ventricular systolic pressure in type 2 diabetic rats (71).

### 4.8 Drug-induced cardiotoxicity

Isoproterenol (ISO), a β-adrenergic agonist used clinically for the treatment of conditions such as bronchial asthma, cardiac arrest, shock, and hypotension, can lead to myocardial energy depletion, increased myocardial oxygen consumption, and excessive release of oxygen free radicals, ultimately resulting in ischemic necrosis of the myocardium when administered in excess (147, 148). In the aforementioned sections, we discussed the therapeutic effects of MT and OMT on ISO-induced heart failure (68, 133). Clinical pharmacological research indicated that MT and OMT were effective in treating ISO-induced myocardial injury in rats. Oral administration of MT in rats increased the activity of SOD, CAT, and GSH-Px in plasma and myocardial tissue, reduced the content of the lipid peroxidation product malondialdehyde, inhibited the expression of the eNOS inhibitor ADMA, restored the expression of damaged ventricular Akt and eNOS proteins, and also restored Gsk3β activity (46, 47).

Doxorubicin (DOX), a broad-spectrum antitumor agent clinically utilized for various cancers, is substantially limited in its clinical application due to pronounced cardiotoxic side effects. It is reported that DOX's cardiotoxicity commonly manifests as dilated cardiomyopathy, accompanied by increased cardiomyocyte apoptosis, which can lead to severe heart failure (149). Studies indicated that DOX induced myocardial cell oxidative stress, characterized by increased CK-MB and LDH activities and ROS levels, and decreased SOD, CAT, and GSH-Px levels, resulting in myocardial tissue damage and apoptosis. These effects could be effectively reversed by MT and OMT (43, 150). Additionally, Hu et al. (43) found that the antioxidative effects of MT were associated with activating the AMPKα/UCP2 signaling pathway. Activation of this pathway reduced mitochondrial Ca2 + influx, thereby inhibiting ROS synthesis and mitochondrial death. In DOX-treated H9c2 cardiomyocytes, MT enhanced mitochondrial Na + -K + -ATPase and Ca2 + ATPase activities, improved mitochondrial membrane potential (MMP), and reduced apoptosis rates (42).

Aldosterone (ALD) induced myocardial cell injury through various mechanisms, including the phosphorylation of the MAPK signaling pathway (151). JNK protein, as a member of the MAPK family, played a crucial role in cellular stress responses. OMT restored normal morphology in cardiomyocytes by inhibiting the increase in ALD-induced p-JNK expression levels, without suppressing JNK mRNA expression. This suggested that OMT may only inhibit JNK phosphorylation without preventing its synthesis (76, 152). Moreover, OMT mitigated ALD-induced proliferation and migration of cardiac fibroblasts, and reduced levels of α-SMA, type I collagen, type III collagen, and CTGF, thereby attenuating cardiac fibrosis. Mechanistically, this regulatory effect relied on the dissociation of Nrf2-Keap1 and the nuclear translocation of Nrf2, a key modulator of antioxidant action (153).

Viral and bacterial infections can lead to shock and infectious myocarditis, ultimately causing symptoms such as difficulty breathing and acute heart failure (154). Sepsis, a systemic inflammatory response syndrome caused by the invasion of pathogens such as bacteria, identifies the heart as a primary target organ for induced organ dysfunction (155). OMT exhibited anti-inflammatory properties, attenuating the expression of NF-κB, TNF-α, and lipopolysaccharide-binding protein in the myocardial tissue of septic rats, as well as the activation of the upstream regulatory MAPK pathway, thereby reducing heart rate and improving ventricular pressure changes in infected rats (79). Reports suggested that miR-106b-5p could alleviate inflammation-induced cardiac dysfunction (156). With MT administration, the expression of miR-106b-5p was upregulated in cardiomyocytes, and the expression of its sponge protein PTENP1 was downregulated (45).

## 5 Clinical trials and safety

Presently, there is a scarcity of clinical application reports for MT and OMT in cardiovascular and cerebrovascular diseases. *Sophora flavescens* Ait. (SFA) is the principal source of MT and OMT. Formulations containing SFA, as well as traditional Chinese medicine compounds, have demonstrated beneficial effects in treating cardiovascular diseases. Clinical data from 59 breast cancer patients, showing cardiotoxicity due to chemotherapy, demonstrated that adjunct treatment with Compound SFA Injection effectively reduced myocardial enzyme levels and the incidence of electrocardiogram abnormalities (157). Modified Huanglian Wendan Decoction, comprising SFA and 12 additional Chinese herbs, had been clinically proven to effectively alleviate clinical symptoms in patients with ventricular premature contractions caused by hypertensive left ventricular hypertrophy, reducing the frequency of such contractions. It also normalized heart rate variability, thereby improving cardiac autonomic dysfunction (158). Mengshi Kushen Huanglian Decoction. a traditional Chinese medicine formula consisting of lapis chloriti, SFA, and *Coptis chinensis* Franch., had demonstrated significant efficacy in reducing the occurrence of ventricular premature beats and alleviating associated symptoms, as shown in a clinical trial (159). Furthermore, another clinical study had shown that the traditional Chinese medicine formula Sanshen Gansong Decoction, containing SFA among other herbs, could reduce triglyceride, total cholesterol, and ox-LDL levels in patients with coronary heart disease experiencing ventricular premature beats. It also revealed no adverse effects on liver function, renal function, or blood pressure during clinical use (160).

The survey indicated that there were 138 marketed drugs based on MT and OMT, primarily available as injectables. These were generally safe for clinical use, with toxicity manifesting only at elevated concentrations (http://www.da.yaozh.com, last accessed on 26/03/2024). MT at a concentration of 140 mg/ml could reduce cell viability, enhance the protein expression of CYP2A6, CYP2B6, and CYP3A4, and decrease levels of LDH and AST, indicating hepatotoxicity (161). MT (50, 100, 150, 200, and 250 mg/L) exhibited teratogenic and lethal effects on zebrafish embryos in a concentration-dependent manner, with EC50 and LC50 values of 145 and 240 mg/L, respectively (162). Additionally, various acute toxicity tests involving intravenous and oral administration in mice indicated that the LD50 of OMT was approximately 200 mg/kg. Symptoms in mice included increased respiration, reduced mobility, decreased activity, reduced levels of hepatic glutathione, and elevated hepatocyte apoptosis rates (163–165).

## 6 Discussion

Synthesizing past research, MT and OMT, as natural medicinal components, exert cardioprotective effects through various pharmacological actions. These include treating myocardial ischemia, cardiomyocyte injury, cardiac fibrosis, ventricular remodeling, heart failure, atherosclerosis, and diabetic cardiomyopathy, among others. MT and OMT feature multi-target, multi-pathway characteristics in cardiovascular disease treatment. MT mitigates cardiomyocyte apoptosis or autophagy by activating the JAK/STAT pathway, regulating the imbalance of pro-apoptotic and anti-apoptotic proteins, as well as inflammatory factors. Both MT and OMT reduce oxidative stress-induced cardiac cell dysfunction, primarily through the mitigation of excessive ROS production or accumulation, involving endoplasmic reticulum stress and ferroptosis responses. The expression of the Nrf2/HO-1 and PI3 K/AKT pathways has been commonly observed during pharmacological interventions. In the treatment of cardiac fibrosis, MT and OMT primarily exhibit an inhibitory effect on the TGF-β1/Smad pathway, thereby preventing the formation and deposition of the extracellular matrix (ECM) and the expression of inflammatory factors. The MAPK/ERK1/2 and Notch signaling pathways also play significant roles in this context. Additionally, they prevent atherosclerosis by eliminating inflammation, oxidative responses, and excessive proliferation in vascular cells. They also maintain normal action potential changes by regulating intracellular ion channels (K^+^, Ca^2+^) in cardiomyocytes, thus normalizing abnormal heart rates.

In current research, MT and OMT exhibit low bioavailability. Studies on modern formulations offer numerous benefits. Developing new formulations of MT and OMT can enhance their solubility and stability in the gastrointestinal tract. For instance, encapsulation in nano-carriers such as Covalent Organic Frameworks (166), liposomes (167), chitosan (168, 169), poly (lactide-co-glycolide) microspheres (170), microemulsions (171), and polymersomes (172) can effectively overcome their limitations. Additionally, the combination of MT and OMT with other drugs may improve their pharmacokinetic parameters (26).

Current research indicates that MT and OMT are generally considered safe at recommended doses. However, high doses may lead to adverse reactions such as indigestion and liver damage. Therefore, patients should consult healthcare professionals prior to using MT or OMT for cardiovascular diseases to ensure safety and optimal therapeutic outcomes.

In summary, MT and OMT are two promising natural alkaloid compounds for treating cardiac diseases, owing to their multifactorial actions such as anti-inflammatory, antioxidant, anti-apoptotic, and ion balance regulation. However, the discovery of their cardioprotective effects is primarily based on animal or cellular experimental models, with most corresponding clinical studies focusing on herbal formulations containing these components. This inevitably introduces certain objective limitations, insufficient to demonstrate their practicality and efficacy in clinical applications. Therefore, further research and clinical trials are necessary to better understand their mechanisms of action, optimize treatment regimens, and ensure safety. Future studies should also focus on exploring the optimal dosage of MT and OMT, their long-term effects, and the potential for combination with other therapeutic approaches.

## Glossary

ADMA: Asymmetric dimethylarginine
ATF6: Activating transcription factor 6
Atg5: Autophagy protein 5
AngⅡ: Angiotensin II
AB: Aortic banding
AGEs: advanced glycation end products
ACTA2: actin alpha 2
ARE: Activation of antioxidant response element
APD: Action potential duration
AIF: Apoptosis-inducing factor
AST: Aspartate amino transferase
ATP: adenosine triphosphate
AMP: adenine nucleotides
ACE: Angiotensin converting enzyme
Bcl-xl: B cell lymphoma extra-large
Bcl-2: B cell lymphoma-2
BNP: Brain natruretic peptide
CK-MB: Creatine kinase-MB
CTnT: Cardiac troponin T
CTnI: Cardiac troponin I
CVDs: Cardiovascular diseases
CAT: Catalase
CMECs: Cardiac microvascular endothelial cells
Cx43: connexin 43
CPK: creatine phosphokinase
COX-2: Cyclooxygenase-2
cTn-I: cardiac troponin I
CHOP: C/EBP-homologous protein
CTGF: Connective tissue growth factor
DRG: Dorsal root ganglia
DDAH2: Dimethylarginine dimethyllaminohydrolase 2
DOX: Doxorubicin
ERS: Endoplasmic reticulum stress
eNOS: Endothelial nitric oxide synthase
ECM: Extracellular matrix
FKBP12.6: FK506-binding protein 12.6
GSH-Px: Glutathione peroxidase
GPX4: Glutathione peroxidase 4
GRP78: Glucose-regulated protein
GHb: Glycosylated hemoglobin
GLP-1: Glucagon-like peptide-1
GLUT-4: Glucose transporter-4
Gsk3β: glycogen synthase kinase 3β
HSPs70: Heat shock proteins 70
HOTAIR: HOX transcript antisense RNA
HCSMCs: Human coronary artery smooth muscle cells
HUVEC: Human umbilical vein endothelial cell
HASMCs: Human aortic smooth muscle cells
HDL-c: High density lipoprotein cholesterol
HO-1: heme oxygenase-1
IRE1α: Inositol-requiring enzyme 1α
I/R: Ischemia-reperfusion
ISO: Isoprenaline
ICAM-1: Intercellular adhesion molecule-1
Ito: Transient outward potassium Current
ICa-L: L-type Ca2 + channels
IK1: Inwardly rectifying potassium current
IKM3: M3-receptor-activated delayed rectifier K + current
JAK: Janus kinase
LDH: Lactate dehydrogenase
LDL-c: Low density lipoprotein cholesterol
MT: Matrine
MYH11: Mooth muscle myosin heavy chain
Mfn2: Mitofusin 2
MAPK: Mitogen-activated protein kinase
NLRP3: NLR family pyrin domain containing 3
NEFA: Non-esterified fatty acid
Nrf2: Transcription of nuclear factor erythroid 2-like 2
OMT: Oxymatrine
ox-LDL: Low-density lipoprotein
Papp: Apparent permeability
P-gp: P-glycoprotein
PERK: protein kinase RNA-like endoplasmic reticulum kinase
Poldip2: polymerase delta interacting protein 2
RPS5: Ribosomal protein S5
p70S6 K: 70 kDa Ribosomal Protein S6 Kinase 2
PTENP1: pseudogene of PTEN tumor suppressor
RyR-2: Ryanodine receptor type 2
RAAS: Renin-angiotensin-aldosterone system
SCG: Superior cervical ganglia
STAT: Signal transducer and activator of transcription
SOD: Superoxide dismutase
TGF-β1: Transforming growth factor beta 1
TC: Total cholesterol
TG: Triglyceride
TLR-4: Toll-like receptor-4
UCP2: Uncoupling protein 2
VSMC: Vascular Smooth Muscle Cell
VCAM-1: vascular cell adhesion molecule-1
VASP: Vasodilator-Stimulated Phosphoprotein
XO: Xanthine oxidase
α-SMA: α-smooth muscle actin
β3-AR: β3-adrenoreceptor
